# Novel Recovery of Nano-Structured Ceria (CeO_2_) from Ce(III)-Benzoxazine Dimer Complexes via Thermal Decomposition

**DOI:** 10.3390/ijms12074365

**Published:** 2011-07-05

**Authors:** Chatchai Veranitisagul, Attaphon Kaewvilai, Sarawut Sangngern, Worawat Wattanathana, Songwut Suramitr, Nattamon Koonsaeng, Apirat Laobuthee

**Affiliations:** 1 Department of Materials and Metallurgical Engineering, Faculty of Engineering, Rajamangala University of Technology Thanyaburi, Pathumthani 12110, Thailand; E-Mail: veranitisagul.c@gmail.com; 2 Department of Materials Engineering, Faculty of Engineering, Kasetsart University, Bangkok 10900, Thailand; E-Mails: kaewvilai@hotmail.com (A.K.); kindaiji2005@hotmail.com (S.S.); 3 Department of Chemistry, Faculty of Science, Kasetsart University, Bangkok 10900, Thailand; E-Mails: w_worawat@hotmail.com (W.W.); fsciswsm@ku.ac.th (S.S.)

**Keywords:** benzoxazine dimers, cerium complex, ceria, nanoparticles

## Abstract

*N*,*N*-bis(2-hydroxybenzyl)alkylamines, benzoxazine dimers, are the major product produced from benzoxazine monomers on mono-functional phenol by the one step ring opening reaction. Due to the metal responsive property of benzoxazine dimers, in this present work, *N,N*-bis(5-methyl-2-hydroxybenzyl)methylamine (MMD), *N*,*N*-bis (5-ethyl-2-hydroxybenzyl)methylamine (EMD), and *N*,*N*-bis(5-methoxy-2-hydroxybenzyl) methyl amine (MeMD), are considered as novel ligands for rare earth metal ion, such as cerium(III) ion. The complex formed when the clear and colorless solutions of cerium nitrate and benzoxazine dimers were mixed, results in a brown colored solution. The metal-ligand ratios determined by the molar ratio and the Job’s methods were found to be in a ratio of 1:6. To clarify the evidence of the complex formation mechanism, the interactions among protons in benzoxazine dimers both prior to and after the formation of complexes were determined by means of ^1^H-NMR, 2D-NMR and a computational simulation. The single phase ceria (CeO_2_) was successfully prepared by thermal decomposition of the Ce(III)-benzoxazine dimer complexes at 600 °C for 2 h, was then characterized using XRD. In addition, the ceria powder investigated by TEM is spherical with an average diameter of 20 nm.

## 1. Introduction

Ceria, cerium(IV) oxide, or cerium oxide (CeO_2_) with a fluorite crystal structure has been considered as a material for applications in solid oxide fuel cell, catalysts, hydrogen storage materials, ultraviolet absorbers, *etc.* [1–3]. The preparation of CeO_2_ for diverse applications, includes various methods, such as, co-precipitation, sol-gel, hydrothermal method, thermal decomposition, *etc.* [4–10]. However, a technique which provides several advantages over traditional methods in terms of homogeneity and purity of products, low processing temperatures, and the ease to control the size, shape, and distribution of ceramic particles remains under development.

For the past few years, it was found that *N*,*N*-bis (2-hydroxybenzyl) alkylamines, the major product from the single step ring opening reaction of benzoxazine monomer on mono-functional phenol, exhibit an excellent responsive property to various metal ions [11–15]. However, the complex solutions attained as a by-product are usually discarded. In a related work, the complex method to prepare the high purity and homogeneity ceria for application as the solid support catalyst for methane steam reforming, and electrolyte for solid oxide fuel cells, has been focused. By means of such a method to prepare the metal complexes, the conditions for the complexation can be easily varied. The environment for the completion of the reaction can thus be reduced to room temperature, while general solvents, for instance alcohol, hexane, dioxane, *etc.* can be employed. Moreover, specific and expensive apparatuses are not required.

Based on the molecular structures and the excellent properties of benzoxazine dimers, in this present work, benzoxazine dimers as novel ligands for Ce(III) ion were proposed. By means of the molar ratio and the Job’s methods, the qualitative and quantitative studies of the complexation of benzoxazine dimers and Ce(III) ion were studied. To investigate interaction of benzoxazine dimers toward Ce(III) ion, free benzoxazine dimers and post complexation properties were studied by means of ^1^H-NMR and 2D-NMR (NOESY). In addition, the complexes between Ce(III) and benzoxazine dimers were further applied as precursors for the preparation of nano-structured ceria powder. Thermal properties of the complexes were investigated by TGA. The obtained ceria powders were also studied by XRD, BET and TEM.

## 2. Results and Discussion

### 2.1. Complexation of Benzoxazine Dimers and Ce(III) Ion

**Figure 1:**
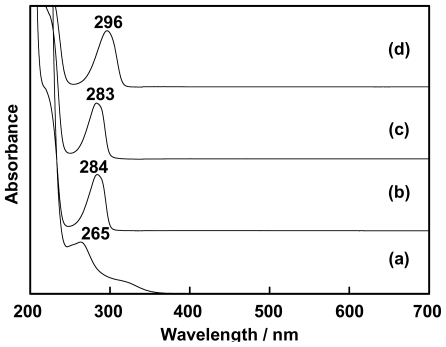
UV-Vis absorption spectra of ethanolic solutions of (**a**) cerium(III) nitrate; (**b**) MMD; (**c**) EMD, and (**d**) MeMD.

The clear and colorless solutions of benzoxazine dimers and cerium(III) nitrate hexahydrate [Ce(NO_3_)_3_·6H_2_O] were analyzed for their characteristics with a UV-Visible spectrometer. The maximum absorption peaks in the ultraviolet region of cerium(III) nitrate, MMD, EMD and MeMD were 265, 284, 283 and 296 nm, respectively (Figure 1).

**Figure 2:**
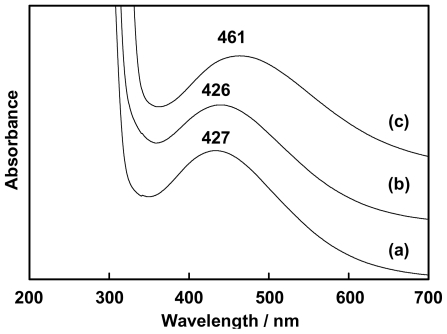
UV-Vis absorption spectra of complexes in ethanol (**a**) Ce(III)-MMD; (**b**) Ce(III)-EMD and (**c**) Ce(III)-MeMD.

The brown solutions of cerium(III) nitrate and benzoxazine dimers mixtures confirmed the complex formation and showed λ_max_ at 427, 426 and 461 nm for Ce(III)-MMD, Ce(III)-EMD and Ce(III)- MeMD complexes, respectively (Figure 2).

**Figure 3:**
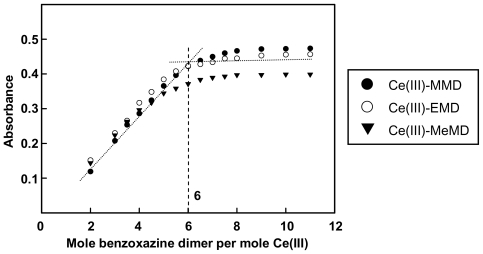
Molar ratios’s plot for Ce(III)-benzoxazine dimers in ethanol.

**Figure 4:**
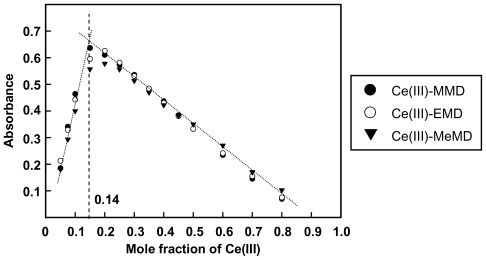
Job’s plot for Ce(III)-benzoxazine dimers in ethanol.

Figures 3 and 4 show the plots of Ce(III)-ligand complexes ratios studied at their λ_max_. The results obtained from the molar ratio and the Job’s method were in good agreement for the ratio of Ce(III) to benzoxazine dimer of 1:6. The complex formations were also studied in methanol, ethanol, and propan-1-ol solvents and the results indicate a ratio of 1 to 6 for the Ce(III)-benzoxazine dimer.

**Figure 5:**
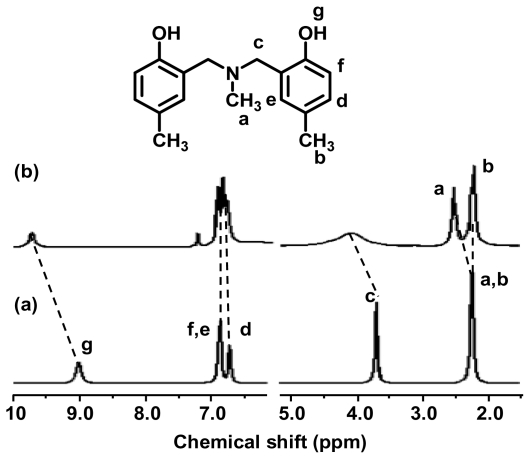
^1^H-NMR spectra of (**a**) MMD and (**b**) Ce(III)-MMD complex in CDCl_3_.

The interactions between benzoxazine dimers and Ce(III) ion were investigated by ^1^H-NMR spectroscopy. The ^1^H-NMR spectra of free MMD ligand and Ce(III)-MMD complex were compared (Figure 5). It was determined that the peak of methylene protons (position c) significantly shifted from 3.69 to 3.94 ppm and changed from a sharp peak to a broad crest. Moreover, the singlet peak (2.23 ppm) of all-CH_3_ groups (positions a and b) shifted and split to doublet peaks (2.22 and 2.39 ppm). The shifting and splitting of the peaks indicated that the environment of protons at aza (N-CH_3_) and methylene (N-CH_2_-) was subject to change as the electron paired donor on the nitrogen atom coordinates to the Ce(III) ion, so that the electrons of aza (N-CH_3_) and methylene (N-CH_2_-) groups were withdrawn, resulting in a decrease in electron density of the aforementioned protons. Consequently, the chemical shifts of protons on aza and methylene groups were shifted to the low fields. Moreover, the singlet peak of hydroxyl protons (position g) at 9.05 ppm was shifted to a lower field of 9.75 ppm that might be due to the disappearance of the intramolecular hydrogen bond between a hydroxyl group and aza group (O-H▪▪N) in benzoxazine dimer. This implied that on complexation, the hydrogen bond in benzoxazine dimer was eliminated to allow the lone paired electron of nitrogen atom coordinating to Ce(III) ion. The complex formation of EMD and MeMD benzoxazine dimers showed similar results, which indicates benzoxazine dimer derivatives are monodentate ligands and only nitrogen atom at aza group (N-CH_3_) is a coordinating atom.

**Figure 6:**
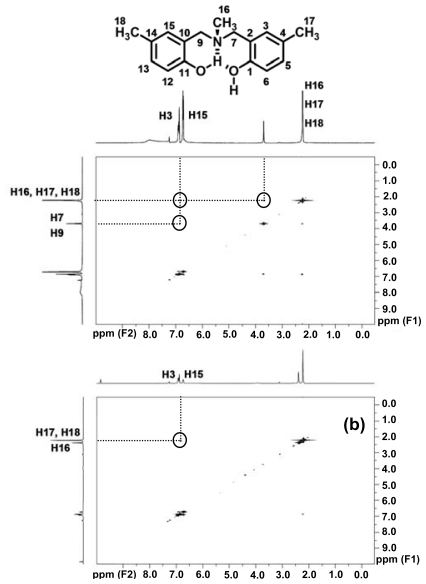
^1^H-^1^H NOESY spectra of (**a**) MMD; (**b**) Ce(III)-MMD complex in CDCl_3_.

The interaction between benzoxazine dimers and Ce(III) ion was also studied by means of the ^1^H-^1^H NOESY technique to investigate the complex formation. The ^1^H-^1^H NOESY spectra (Figure 6) indicate that the methylene protons (H7 and H9) do not reveal the interaction of the aromatic protons (H3 and H15) and methyl protons (H16, H17 and H18) upon the formation of the complex. In addition, the interaction between methyl protons of aza group (H16) and aromatic protons (H3 and H15) disappeared while the interaction between methyl protons on aromatics (H17 and H18) and aromatic protons (H3 and H15) remained evident. During complexation, conformations of benzoxazine dimers might generate the suitable structures for nitrogen atom to interact with the Ce(III) ion. The environment of protons (H7, H9 and H16) connected to the nitrogen atom was, therefore, changed after complexing and resulted in the disappearance of the interactions as shown in Figure 6. These results indicate that the complex was generated by the coordinated covalent bond between the Ce(III) ion and the nitrogen atom of benzoxazine dimers. However, before and after complexing, the hydroxyl protons show no evidence of the interaction with other protons. Similarly, EMD and MeMD yield results which imply the formation of the complex by the interaction between the nitrogen atom and the Ce(III) ion.

**Figure 7:**
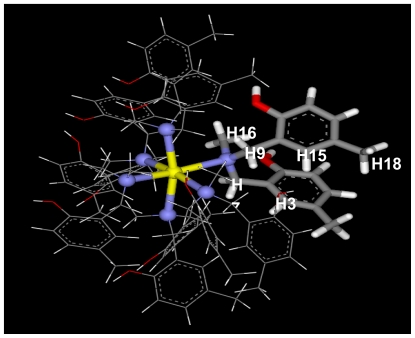
Orientation of Ce-MMD complex.

**Figure 8:**
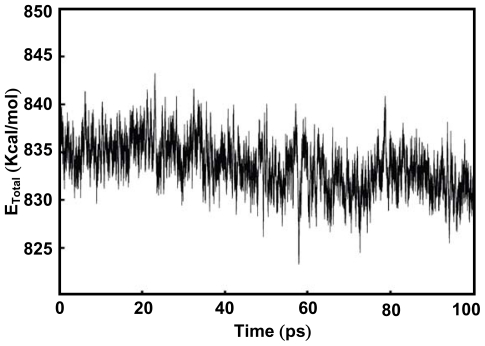
Graph of potential energy *vs.* time during geometry optimization.

To confirm these results, the computational simulation of the Ce-MMD complex was studied. The feasible structure of the Ce-MMD complex is shown in Figure 7 and the deviation in potential energy is plotted against time in Figure 8. The starting structure of this simulation was based on NMR results. The potential energy plots indicated that Ce-MMD complex is well equilibrated after 100 ps of the simulation.

The Ce(III) ion showed specific binding to the N-atom of the MMD ligand along the octahedral structure during the optimization using molecular mechanics. A molecular dynamics simulation was carried out at a constant temperature of 300 K. As expected, the Ce(III) ion was attracted to the N-atom of the octahedral with a stable trajectory. This geometry proved that under room temperature Ce(III) ion specifically binds to the N-atom of MMD molecule in octahedral complex.

In theoretical aspect, the orientation of complex was in agreement with the experimental results. ^1^H-^1^H NOESY spectrum of cerium complex shows no longer correlation between the methylene protons (H7 and H9) and aromatic protons (H3, H15, H16, H17 and H18) corresponding to configuration of complex from computational study.

### 2.2. Preparation of Ceria (CeO_2_) from Ce(III)-Benzoxazine Dimer Complexes

**Figure 9:**
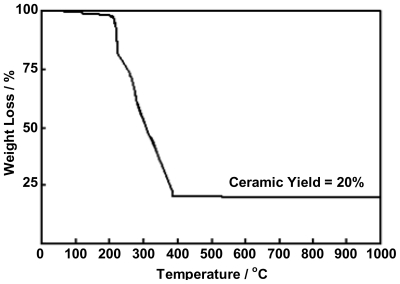
TGA thermogram of Ce(III)-MMD complex.

To obtain the CeO_2_ powder product, the Ce(III)-benzoxazine dimer complexes were calcined to remove the organic contents. The appropriate temperature for calcination of Ce(III)-benzoxazine dimer complexes were determined by means of TGA. The Ce(III)-MMD complex exhibited decomposition on heating as revealed in the three-step weight loss patterns (Figure 9). The first mass loss involved the decomposition of the organic ligand and this occurred at temperatures from 100 to 250 °C. During this step, volatiles and char were generated. As shown in the second mass loss, the obtained char was continuously oxidized by heating from 250 to 380 °C. The slight mass loss observed in the 380 to 530 °C temperature range was ascribed to the burning of the residual organic contents. At temperatures in excess of 530 °C, no mass loss was found, that indicates an appropriate calcination temperature for the preparation of the powder product should be started at 600 °C. In addition, the further complexes, Ce(III)-EMD and Ce(III)-MeMD also exhibited similar decomposition patterns as those of Ce(III)-MMD. The final mass losses of the complexes were established at approximately 20% (Figure 9). Based on the TGA results, all of the Ce(III)-benzoxazine dimer complexes were, therefore, calcined at 600 °C for 2 h to attain the characteristic pale yellow color of powder products.

**Figure 10:**
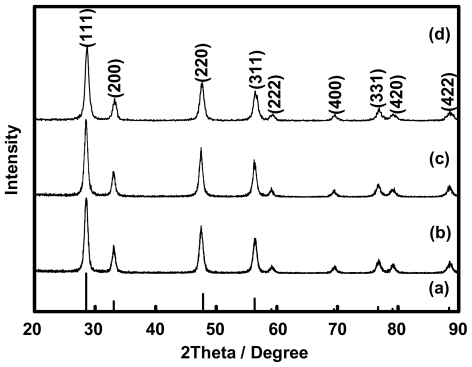
XRD patterns of ceria derived from Ce(III)-benzoxazine dimer complexes (**a**) JCPDS No. 34–0394 (**b**) Ce(III)-MMD (**c**) Ce(III)-EMD and (**d**) Ce(III)-MeMD.

By the applying the XRD technique, the calcined powders derived from all of the Ce(III)-benzoxazine dimer complexes displayed the reflection peaks which correlated to (111), (200), (220), (311), (222), (400), (331), (420) planes located at 2θ = 28.535°, 33.080°, 47.495°, 56.348°, 59.102°, 69.427°, 76.710°, 79.073°, and 88.447°, respectively (Figure 10). All of the reflection peaks are in agreement with the face-centered cubic, CeO_2_ (JCPDS No. 34-0394), which identified the calcined to be ceria with the fluorite structure.

Table 1 shows the physical characteristics of the ceria products obtained. It appears evident that all of ceria derived from all complexes do not significantly differ in the specific surface area (*S*_BET_), the particle size (*D*_BET_), including the crystallite size, determined by the application of the Scherrer equation.

**Figure 11:**
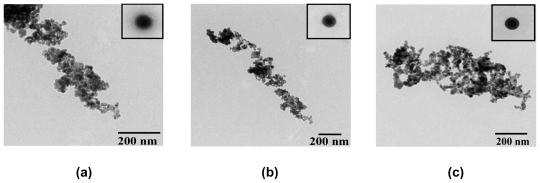
TEM micrographs at 100 kV of ceria nanoparticles derived from Ce(III) benzoxazine dimer complexes (**a**) Ce(III)-MMD (×25.0 k) ; (**b**) Ce(III) EMD (×12.0 k) and (**c**) Ce(III)-MeMD (×20.0 k).

By the application of the Ce(III)-benzoxazine dimer complexes as precursors for the ceria preparation, it was determined that the surface area of ceria increases three-fold compared to previous studies in which triethanolamine was applied as a ligand. [16] To confirm the size and shape of ceria, TEM was performed. Most particles obtained from all complexes were spherical, with an average diameter of 20 nm as shown in Figure 11. In addition, the results indicate that the type of benzoxazine dimers does not affect the morphology and the particle size of ceria.

## 3. Experimental Section

### 3.1. Chemicals

Paraformaldehyde was purchased from Sigma (U.S.A.). 4-Methoxyphenol, 4-ethylphenol, *p*-cresol, methylamine (40% w/v in water), potassium hydroxide, and sodium sulfate anhydrous were purchased from Fluka Chemicals (Buchs, Switzerland). Cerium(III) nitrate hexahydrate [Ce(NO_3_)_3_·6H_2_O, 99.5% purity] was purchased from Acros Organics. Ethanol, methanol, propan-1-ol, sodium hydroxide, and diethylether were the products of Ajax chemicals (Australia). All chemicals were analytical grade and used as received.

### 3.2. Instruments

The UV-Visible spectra and the absorbance of all mixed solutions were measured by the application of a Perkin Elmer Lambda35 UV-Vis spectrometer over a wavelength of 200 to 700 nm. The complexes were characterized by Fourier transform proton nuclear magnetic resonance (^1^H-NMR) spectrometer (Varian Mercury-400 spectrometer) with CDCl_3_ as a solvent.

The decomposition aspects and weight losses of all the cerium complexes were evaluated with a TGA analyzer (Perkin-Elmer TGA 7). Samples (10 mg) were loaded in an alumina crucible and heated at the heating rate of 5 °C/min under the air flow. The thermograms were recorded at a temperature range of 50–1000 °C.

The calcined ceria products were investigated by X-ray diffraction (XRD) using a Bruker D8-Advance X-ray diffractrometer with CuK_α_ radiation. Diffraction patterns were recorded over a range of 2θ angles from 20 to 90 degrees in a step-scanning mode (0.02° steps with a step counting time of 2 s). The crystalline phase was identified from the Joint Committee on Powder Diffraction Standard (JCPDS) file No. 34-0394.

Specific surface area (S_BET_) measurements were carried out by employment of the Brunauer-Emmett-Teller (BET) analysis by nitrogen adsorption isotherms at 77 K with a Micromeritics ASAP 2020 surface analyzer and a value of 0.162 nm^2^ for the cross section of the nitrogen molecule. Samples were degassed at 350 °C under a nitrogen vacuum for 20 h prior to measurement.

The *S*_BET_ measurements were translated into the average particle size (D_BET_) accordance with formula; *D*_BET_ = 6000/(*d*_th_ × *S*_BET_). Where D_BET_ is the average particle size (nm), S_BET_ is the specific surface area (m^2^/g), and *d*_th_ is the theoretical density of the solid solution oxide (7.211 g/cm^3^).

The morphology of the obtained ceria powders observed by transmission electron microscope (TEM) was taken at an accelerating voltage current of 100.0 kV by Hitachi H-7650 (Hitachi High-Technology Corporation, Japan).

### 3.3. Complexation of Benzoxazine Dimers and Ce(III) Ion

**Figure 12:**
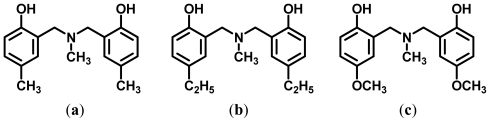
Structures of (**a**) MMD (**b**) EMD and (**c**) MeMD.

Benzoxazine dimers (Figure 12): *N*,*N*-bis(5-methyl-2 hydroxybenzyl)methylamine (MMD), *N*,*N*-bis(5-ethyl-2-hydroxybenzyl)methylamine (EMD), and *N*,*N*-bis(5-methoxy-2-hydroxybenzyl) methylamine (MeMD), were prepared as reported elsewhere [11] and employed as ligands for cerium(III) ion. The ethanolic solutions of cerium(III) nitrate and three benzoxazine dimers (MMD, EMD, and MeMD) were individually prepared with an equimolar concentration of 1.00 × 10^−4^ M as to study the complex formation by the molar ratio method and the Job’s method.

For the molar ratio method, a series of solutions containing 1.00 mL of cerium(III) nitrate and 1.00 × 10^−4^ M of each benzoxazine dimer (MMD, EMD or MeMD) of variant volumes (2.00, 3.00, 3.50, 4.00, 4.50, 5.00, 5.50, 6.00, 6.50, 7.00, 7.50, 8.00, 9.00, 10.00 and 11.00 mL) were mixed and subsequently adjusted with ethanol as to attain a total volume of 25.00 mL.

For the Job’s method, a series of mixture solutions of Ce(III) ion and each benzoxazine dimer with various Ce(III) ion mole fractions, *X* (*X* = 0.05, 0.08, 0.10, 0.15, 0.20, 0.25, 0.30, 0.35, 0.40, 0.45, 0.50, 0.60, 0.70 and 0.80) were prepared.

The Ce(III)-benzoxazine dimer complexes were dissolved in CDCl_3_ for the ^1^H and ^1^H-^1^H NOESY NMR analysis.

### 3.4. Computational Simulation

All calculations were carried out by using the HyperChem 7.5 [17] molecular modeling software on a Windows XP operating system. Molecular mechanics and molecular dynamics methods were utilized at arriving at the proposed structure. The structure of cerium complex was drawn and optimized using molecular mechanics employing the MM+ force field designed by Allinger for the simulation of most non-biological species [18]. The geometry was optimized without any constraint allowing all atoms, bonds and dihedral angles to change simultaneously. The Polak-Ribiere conjugate gradient algorithm was used to find the minimum to an energy convergence of 0.001 kcal/Å mol^−1^. A 100 ps molecular dynamics simulation was carried out in vacuum under constant temperature condition (300 K). A bath relaxation time of 0.1 ps and a step size of 0.001 ps were used for the structural simulation. The molecular dynamics simulation was used to understand the geometry of Ce-MMD complex.

### 3.5. Preparation of Ceria (CeO_2_) from Ce(III)-benzoxazine Dimer Complexes

All solutions of Ce(III)-benzoxazine dimer complexes were collected and dried over anhydrous sodium sulfate to eliminate water and moisture in the solution. The solvent was then removed by vacuum distillation to obtain the brown solid products. Prior to the conversion of the brown complexes to ceria powder by the calcination process, the composition aspects and weight losses of all complexes were studied by means of thermogravimetric analysis (TGA). To obtain the CeO_2_ powders, all of the complexes were calcined in alumina crucibles at a temperature of 600 °C for 2 h in air. The powders obtained were studied by XRD for phase identification, BET for surface area and TEM for morphology observation.

## 4. Conclusions

This study revealed all of the proposed benzoxazine dimers to be novel ligands for Ce(III) ion. By the molar ratio and the Job’s methods, the metal-ligand ratio of benzoxazine dimers (MMD, EMD and MeMD) and Ce(III) ion was determined to be constant at 6:1 in an ethanolic solution. ^1^H NMR and ^1^H-^1^H NOESY suggested that coordinated atoms of ligand benzoxazine dimers in Ce(III) complexes are composed of a nitrogen atom of the aza-methylene group. In addition, the substituted groups on para positions of benzoxazine dimers (MMD, EMD and MeMD) do not affect the formation of complexes. The possible structure of Ce-MMD complex simulated by Hyperchem was proposed and found to be in agreement with the experimental results.

The pure ceria nanoparticles were successfully prepared from the complexes Ce(III)-benzoxazine dimers by calcinating at 600 °C for 2 h. The obtained particles were spherical with an approximate size of 20 nm. By the application of the aforementioned method, diverse advantages are evident, such as, a simpler reaction which occurs in absence of specific solvents at room temperature. Additionally, ceria with a high surface area are obtained compared with previous ligands, such as triethanolamine. Moreover, Ce-benzoxazine dimer complexes provide several advantages over traditional methods in terms of homogeneity and purity of products, low processing temperatures, and the ease to control the size, shape, and distribution of ceramic particles remains under development. In related works by the authors of this paper, the acquired ceria nanoparticles can be used as the solid support for metal catalysts.

## Supplementary Materials



## Figures and Tables

**Table 1:** Physical characteristic of ceria derived from the Ce(III)-benzoxazine dimer complexes.

Ce(III)-Benzoxazine Dimer Complexes	*S*_BET_ (m^2^/g)	*D*_BET_ (nm)	Crystallite Size (nm)
Ce(III)**-MMD**	60	13.86	25.96
Ce(III)**-EMD**	65	12.79	26.77
Ce(III)**-MeMD**	64	12.99	25.97
