# Supplementary material for: Novel Recovery of Nano-Structured Ceria (CeO2) from Ce(III)-Benzoxazine Dimer Complexes via Thermal Decomposition
Source: Int J Mol Sci. 2011 Jul 5;12(7):4365–77. doi: 10.3390/ijms12074365 (PMC3155356; doi:10.3390/ijms12074365)
Supplement: Supplementary file 1 [file ijms-12-04365-s001.pdf]

Table 1S. Equilibrium structure the Ce-MMD complex calculated by moleculardynamic simulation.

Atoms	Coordinate		
	x axis	y axis	z axis
Ce	-0.359	0.0061	0.7286
N	-1.1528	-2.4503	0.1764
C	-0.5459	-3.5582	1.0907
C	-2.6299	-2.4597	0.1954
C	-0.684	-2.7832	-1.1603
C	-0.6433	-5.0697	0.736
H	0.5138	-3.3346	1.2087
H	-1.0413	-3.53	2.0665
H	-3.1211	-2.5383	-0.5757
H	-2.9492	-1.8619	1.089
C	-3.3731	-3.8132	0.3081
H	-0.8939	-2.0373	-1.8157
H	0.4503	-2.9426	-1.1921
H	-1.1122	-3.6916	-1.4924
C	-1.4961	-5.9525	1.5081
C	0.3442	-5.6923	-0.1461
C	-3.7407	-4.5625	-0.8374
C	-4.031	-4.1808	1.4972
C	-1.4304	-7.3383	1.3152
H	-2.024	-5.5802	2.3499
C	0.4212	-7.078	-0.2448
O	1.2114	-4.837	-0.6453
C	-4.5718	-5.7004	-0.7742
O	-3.1683	-4.1776	-2.0463
C	-4.8543	-5.3234	1.5938
H	-3.7788	-3.6322	2.3242
C	-0.5631	-7.914	0.3744
C	-2.5519	-8.1192	1.9436
H	1.2733	-7.5466	-0.7717
H	1.737	-4.9086	-1.4908
C	-5.2303	-5.9761	0.4301
H	-4.833	-6.2762	-1.6502
H	-3.3486	-4.7397	-2.748
C	-5.4187	-5.6642	2.9412
H	-0.6583	-8.9513	0.2369
H	-2.9793	-7.641	2.8701
H	-3.3688	-8.2222	1.2267

Table 1S. *Cont.*

H	-2.1539	-9.1217	2.1872
H	-6.0423	-6.6687	0.4889
H	-6.2432	-5.0003	3.2347
H	-5.7909	-6.7337	2.9398
H	-4.6938	-5.5964	3.7582
N	0.0716	2.4063	1.6298
C	0.4551	3.3454	0.5041
C	-1.1559	3.0455	2.3637
C	1.2415	2.577	2.5614
C	0.6564	4.8702	0.7742
H	1.3986	3.0773	0.0235
H	-0.2793	3.3364	-0.3015
H	-1.6467	2.3234	3.0354
H	-1.8776	3.3227	1.6301
C	-1.0349	4.3551	3.2586
H	1.1699	3.5811	3.0225
H	1.3253	1.9225	3.4087
H	2.1677	2.5727	2.0286
C	-0.3292	5.7256	0.2483
C	1.9098	5.3607	1.2101
C	-0.5095	4.3899	4.5797
C	-1.8751	5.3981	2.8704
C	-0.106	7.1222	0.304
H	-1.2268	5.3919	-0.1143
C	2.117	6.729	1.2001
O	2.9126	4.5494	1.6645
C	-0.637	5.5351	5.3552
O	0.1318	3.3042	5.0955
C	-2.1012	6.5611	3.634
H	-2.3298	5.3676	1.9421
C	1.0887	7.6275	0.8264
C	-1.1618	8.1274	-0.1201
H	3.0384	7.1316	1.5632
H	3.8113	4.7415	1.7877
C	-1.4246	6.6499	4.8563
H	-0.2005	5.6006	6.2539
H	0.3823	3.2476	6.002
C	-3.0589	7.6105	3.1762
H	1.3347	8.7124	0.8714
H	-1.3782	8.8061	0.7219

Table 1S. *Cont.*

H	-0.7852	8.6525	-0.9656
H	-2.0882	7.5956	-0.4436
H	-1.5107	7.4942	5.4868
H	-3.6562	7.2012	2.3689
H	-3.7907	7.7716	3.9112
H	-2.594	8.5487	2.963
N	-2.3252	0.7405	-0.9149
C	-2.4167	-0.0661	-2.181
C	-3.5076	0.7129	0.0274
C	-2.1912	2.1988	-1.2983
C	-3.3282	0.2553	-3.3446
H	-1.4006	-0.2615	-2.5669
H	-2.6317	-1.0984	-1.8643
H	-3.452	1.6136	0.6436
H	-3.4234	-0.1897	0.6504
C	-4.9353	0.7221	-0.5298
H	-1.3557	2.3837	-1.9945
H	-3.077	2.4427	-1.9186
H	-2.1601	2.8747	-0.4881
C	-4.3638	-0.6566	-3.6994
C	-3.0422	1.3191	-4.2481
C	-5.609	1.8823	-1.0727
C	-5.6868	-0.4976	-0.4392
C	-5.2481	-0.3159	-4.7413
H	-4.6967	-1.4101	-3.0293
C	-3.8479	1.6216	-5.3448
O	-1.8942	2.1008	-4.1066
C	-6.8725	1.7498	-1.671
O	-5.069	3.1979	-0.8552
C	-7.0321	-0.5868	-0.8545
H	-5.2064	-1.2748	0.1092
C	-4.9732	0.8302	-5.52
C	-6.382	-1.172	-5.1411
H	-3.7154	2.3962	-6.0027
H	-1.7522	2.9327	-4.5869
C	-7.5423	0.5247	-1.5435
H	-7.3781	2.5151	-2.2343
H	-5.5045	4.0116	-1.0983
C	-7.683	-1.9232	-0.7663
H	-5.5723	1.1012	-6.3119

Table 1S. *Cont.*

H	-7.1844	-0.6477	-5.7858
H	-6.0902	-2.1164	-5.7051
H	-7.0125	-1.5237	-4.2941
H	-8.5391	0.4029	-1.9957
H	-7.7588	-2.4263	-1.7891
H	-7.0877	-2.6081	-0.1208
H	-8.776	-1.8204	-0.4218
N	1.7092	-1.0389	1.8674
C	1.3064	-1.7326	3.0872
C	2.4552	-1.988	0.9115
C	2.5826	0.0705	2.1996
C	2.0531	-2.807	3.9013
H	1.1776	-1.0813	3.8768
H	0.3829	-2.2374	2.8623
H	2.8968	-1.4065	0.1636
H	1.7657	-2.6501	0.449
C	3.6251	-2.8419	1.38
H	2.5119	0.8283	1.4213
H	2.2269	0.4343	3.155
H	3.6522	-0.2287	2.32
C	1.4702	-4.1083	4.0259
C	3.2817	-2.5627	4.5609
C	4.9693	-2.367	1.4035
C	3.4759	-4.229	1.5249
C	2.1418	-5.1696	4.7181
H	0.6197	-4.3647	3.4826
C	3.8648	-3.5831	5.363
O	3.8992	-1.3773	4.6115
C	6.0832	-3.1894	1.7991
O	5.273	-1.223	0.8504
C	4.5527	-5.0762	1.8999
H	2.5396	-4.6387	1.4681
C	3.382	-4.8692	5.3041
C	1.5579	-6.5561	4.7348
H	4.7411	-3.3371	5.7742
H	4.6097	-1.1552	5.0855
C	5.8931	-4.556	2.1028
H	7.0548	-2.8234	1.9725
H	6.0934	-0.8859	0.6928
C	4.3526	-6.5273	2.1982

Table 1S. *Cont.*

H	3.9704	-5.5901	5.8463
H	0.6957	-6.65	4.0799
H	2.3153	-7.3239	4.3553
H	1.2849	-6.6497	5.8407
H	6.7679	-5.1508	2.3208
H	4.8503	-7.1723	1.4908
H	4.8548	-6.7984	3.1264
H	3.2933	-6.8004	2.1482
N	1.412	0.4776	-1.2353
C	1.4387	-0.7427	-2.0261
C	1.0548	1.5797	-2.2035
C	2.7863	0.9041	-0.8188
C	2.2858	-0.9402	-3.3108
H	1.7032	-1.5802	-1.369
H	0.418	-0.974	-2.3252
H	0.5195	2.3985	-1.6795
H	0.3543	1.0344	-2.8574
C	2.0147	2.282	-3.1873
H	3.5629	1.1619	-1.5794
H	2.7379	1.7881	-0.2249
H	3.3038	0.1648	-0.2002
C	1.5162	-1.2432	-4.4717
C	3.71	-1.0718	-3.3725
C	2.863	3.3726	-2.8454
C	2.0977	1.8803	-4.536
C	2.1797	-1.6034	-5.682
H	0.4351	-1.0251	-4.3909
C	4.3094	-1.2623	-4.6117
O	4.3541	-1.187	-2.1837
C	3.6949	4.016	-3.8071
O	2.9698	3.8828	-1.5649
C	2.8931	2.509	-5.4839
H	1.4828	1.1155	-4.9198
C	3.5671	-1.5623	-5.7437
C	1.4296	-1.7187	-6.9554
H	5.3409	-1.4888	-4.6902
H	5.3373	-1.0367	-2.1147
C	3.6766	3.625	-5.1711
H	4.2711	4.7869	-3.4966
H	3.5164	4.6176	-1.2975

Table 1S. *Cont.*

C	2.6176	2.0663	-6.9147
H	4.0924	-1.7505	-6.6432
H	0.4021	-1.986	-6.7935
H	1.5602	-0.7896	-7.5798
H	1.8801	-2.4968	-7.5175
H	4.3904	3.9368	-5.8874
H	3.3372	2.5501	-7.6454
H	2.8419	1.007	-6.9773
H	1.6353	2.3727	-7.2015
N	-1.6779	-0.3218	3.0503
C	-3.052	0.4496	2.9507
C	-1.9609	-1.7061	3.4903
C	-0.879	0.3287	4.1129
C	-3.6704	1.3513	4.0673
H	-2.8389	1.0947	2.1481
H	-3.8472	-0.1734	2.5859
H	-1.0293	-2.1032	3.9438
H	-2.2783	-2.2352	2.6472
C	-2.9225	-2.1149	4.6296
H	-0.0001	0.7712	3.6545
H	-1.4094	1.1075	4.6911
H	-0.4457	-0.3731	4.7984
C	-4.4154	2.3928	3.4949
C	-3.7576	1.1264	5.4694
C	-2.4493	-2.6368	5.8459
C	-4.2688	-2.0657	4.4505
C	-5.1989	3.26	4.3279
H	-4.33	2.6311	2.418
C	-4.4422	1.9839	6.3132
O	-3.0686	0.149	6.0429
C	-3.317	-3.0847	6.8711
O	-1.1353	-2.8287	6.05
C	-5.1815	-2.5178	5.4247
H	-4.6595	-1.7119	3.5118
C	-5.0255	3.139	5.716
C	-5.8869	4.4872	3.8142
H	-4.4266	1.846	7.3459
H	-2.9848	0.1599	6.9512
C	-4.6995	-3.1213	6.6322
H	-2.9176	-3.623	7.7223

Table 1S. *Cont.*

H	-0.7743	-3.1403	6.8745
C	-6.6421	-2.5217	5.2062
H	-5.5241	3.8169	6.4443
H	-6.3159	5.1175	4.5594
H	-5.0614	5.0213	3.2538
H	-6.6226	4.2523	3.0402
H	-5.4177	-3.4411	7.351
H	-7.1447	-2.2061	6.0992
H	-6.7517	-1.8871	4.3709
H	-7.0898	-3.4666	5.0625
